# Concurrent Changes of Brain Functional Connectivity and Motor Variability When Adapting to Task Constraints

**DOI:** 10.3389/fphys.2018.00909

**Published:** 2018-07-10

**Authors:** Grégoire Vergotte, Stéphane Perrey, Muthuraman Muthuraman, Stefan Janaqi, Kjerstin Torre

**Affiliations:** ^1^EuroMov, Université de Montpellier, Montpellier, France; ^2^Movement Disorders and Neurostimulation, Biomedical Statistics and Multimodal Signal Processing Unit, Focus Program Translational Neuroscience (FTN), Department of Neurology, Johannes Gutenberg University, Mainz, Germany; ^3^LGI2P, Institut Mines Télécom-Ecole des Mines d'Alès, Alès, France

**Keywords:** adaptability, fNIRS, modularity, fractal properties, tapping

## Abstract

In behavioral neuroscience, the adaptability of humans facing different constraints has been addressed on one side at the brain level, where a variety of functional networks dynamically support the same performance, and on the other side at the behavioral level, where fractal properties in sensorimotor variables have been considered as a hallmark of adaptability. To bridge the gap between the two levels of observation, we have jointly investigated the changes of network connectivity in the sensorimotor cortex assessed by modularity analysis and the properties of motor variability assessed by multifractal analysis during a prolonged tapping task. Four groups of participants had to produce the same tapping performance while being deprived from 0, 1, 2, or 3 sensory feedbacks simultaneously (auditory and/or visual and/or tactile). Whereas tapping performance was not statistically different across groups, the number of brain networks involved and the degree of multifractality of the inter-tap interval series were significantly correlated, increasing as a function of feedback deprivation. Our findings provide first evidence that concomitant changes in brain modularity and multifractal properties characterize adaptations underlying unchanged performance. We discuss implications of our findings with respect to the degeneracy properties of complex systems, and the entanglement of adaptability and effective adaptation.

## Introduction

The huge ability of the brain to exploit its inherent plasticity to adapt to intrinsic or extrinsic constraints over different time scales is stunning and vital (Bassett et al., 2011; Fallani et al., 2014). Depending on circumstances, adaptability may take the form of robustness against changing conditions as well as the form of innovation and evolvability (Whitacre, 2010; Whitacre and Bender, 2010). In a complementary way the brain allows for preserving a given cognitive-motor performance in the face of tumor growth and resection (Duffau, 2014) as well as diversifying the repertoire of our cognitive-motor behaviors with learning, for example (Bassett et al., 2011; Dayan and Cohen, 2011). While some studies has focused on the precise neuro-physiological mechanisms sustaining the brain's capacity to adapt, others have provided insight into more generic organization principles inherent in complex systems, notably through the assessment of brain network connectivity (e.g., Tononi et al., 1994; McIntosh et al., 1999; Sporns, 2012; Tognoli and Kelso, 2014). From this latter perspective, the brain's functional organization has been conceived as a dynamic balance between functional segregation and integration of subparts of the entire network (Friston, 1994; Tononi et al., 1994; Sporns, 2013). At a given observation scale, the brain network can thus be assessed as a modular organization, modules being defined as clusters that are densely connected within but weakly connected between them (Bullmore et al., 2009; Bassett and Gazzaniga, 2011; Sporns and Betzel, 2016). Moreover, complexity is increased by the dynamic properties of the functional connections within and between modules, which may compose and recompose depending on circumstances. In particular, such connectivity scheme is closely related to degeneracy, a key property characterizing the structure-function relationship in the brain (Tononi et al., 1999; Noppeney et al., 2004). Degeneracy refers to a many-to-one structure-function relationship, with a partial functional overlapping between modules of the network: different parts may perform the same function or specialized functions under circumstances (Edelman and Gally, 2001; Price and Friston, 2002; Whitacre and Bender, 2010). Together, the modular and degenerate properties of network connectivity constitute an essential basis for adaptability, supporting robustness and adaptive changes facing various conditions (Jirsa et al., 2010; Whitacre, 2010; Bassett and Gazzaniga, 2011; Grefkes and Ward, 2014). The variety of the dynamical states or network configurations involved to maintain a given function or performance, whether at rest (Deco et al., 2011) or during a task may thus basically reflect adaptation to changing conditions.

Developing in parallel in a bio-behavioral literature, a significant amount of research focusing on the temporal dynamics of diverse macroscale variables (e.g., heartbeat intervals, Ivanov et al., 2001; force production, Athreya et al., 2012; gait and coordination dynamics, Hausdorff et al., 1996; inter-tapping intervals, Torre and Delignières, 2008) has considered that fractal fluctuations are the hallmark of underlying dynamic complexity and system's adaptability (Ivanov et al., 1998; Gilden, 2001; Ashkenazy et al., 2002; Kello et al., 2010; Manor et al., 2010; Torre and Balasubramaniam, 2011; Delignières and Marmelat, 2013). Notably, a breakdown of the fractal properties in pathological and/or elderly compared to young and healthy subjects has been evidenced repeatedly, supporting the idea that loss of fractal properties can be considered a marker of the general loss of adaptability coming along with aging and disease (Goldberger, 1996; Hausdorff et al., 1996; Peng et al., 2000; Blaszczyk and Klonowski, 2001; Lipsitz, 2002). In particular, in the context of neurological disorders such as Parkinson, Huntington or Alzheimer diseases, research programs have been assessing the diagnostic and/or prognostic (Mäkikallio et al., 2001; Goldberger et al., 2002; Hu et al., 2009) power of fractal properties in sensorimotor variables. Thereby studies have made implicit but strong assumptions on a close relationship between network alterations at the brain level and fractal properties at the effector level. In a complementary vein, the fractal properties of motor variables have been shown sensitive to experimental restriction/augmentation of the sensorial feedbacks available to subject's performance on given tasks (Slifkin and Newell, 1999; Manor et al., 2010; Athreya et al., 2012; Warlop et al., 2013). Finally, the literature has evidenced that fractal properties may be variable within a same time series (multifractal series). Different fractal scaling regimes may apply in an intermittent way to different windows of observation within the series, thus reflecting variations in the system's underlying dynamic organization and exploration of new solutions (e.g., Ivanov et al., 2001, 2004; Nunes Amaral et al., 2001; Hu et al., 2004; Stephen and Anastas, 2011; Dixon et al., 2012; Dutta et al., 2013). In fact, where comparison of the Gaussian properties of any variable of interest may indicate unchanged output across groups or experimental conditions, alterations of its fractal properties often reflect underlying reorganizations in the performing system. To our knowledge, however, the question of whether/to what extent the multiple connectivity patterns forming and reforming in the brain directly spill over into the behavioral outcome remains largely unanswered so far: When the brain adapts facing changing conditions to sustain steady motor-behavioral performance, are the *ad hoc* reorganizations in network connectivity reflected in some distinctive fractal properties of behavioral variability?

In view of the above literature, the degeneracy or intermittency of functional brain networks may be reflected in the multifractal properties at the behavioral level. Imposing constraints by manipulating the feedbacks available to perform a motor task is likely to alter the expression of degeneracy in the motor output. Therefore, the purpose of the study was to bridge levels of observation to establish a direct relationship between degenerate connectivity patterns enabling adaptation at the brain level, and fractal properties as their dynamic signature in the sensorimotor outcome. Herein we consider adaptability as the capacity to maintain a given function or performance despite changing constraints. Thus, a heuristic experimental paradigm should allow us to manipulate the experimental constraints imposed to subjects in a given task without these manipulations affecting their performance, by virtue of the system's capacity to adapt. In this way, we should be able to assess jointly the variety of patterns of brain connectivity that are involved intermittently during task performance, and the dynamic fractal properties of the task variable. Therefore, we used the well-known finger-tapping paradigm (Wing and Kristofferson, 1973), where previous literature has showed that experimental deprivations of visual, auditory, or tactile feedbacks are not such as to alter tapping performance (Aschersleben and Prinz, 1995, 1997; Stenneken et al., 2006; Repp and Su, 2013). Following from the above, we hypothesized that the variety and intermittency of brain networks (degeneracy) involved in the task and the dynamical fractal properties of tapping series would evolve jointly as a function of different conditions of feedback deprivation, while tapping performance should stay invariant.

## Materials and methods

### Participants

Thirty-two healthy volunteers took part in the study (9 women, 23 men, 26.9 ± 6.3 years of age). All participants signed a written informed consent before participating in the study. All participants were right-handed according to the Edinburgh Handedness Inventory (Oldfield, 1971) and reported normal hearing and normal or corrected vision. None showed any sign of neurological disease, nor reported extensive practice in music. All procedures were approved by the local ethics committee (IRB-EM: 1610C, Montpellier). All participants gave written informed consent in accordance with the Declaration of Helsinki for human experimentation.

### Experimental design and procedure

The experimental design was an independent-group design, with the experimental factor being the numbers of sensorial feedbacks the participants were deprived from. Participants were randomized to one of the four following conditions: (i) no feedback deprivation (Control), (ii) deprivation of one feedback, either visual, or auditory, or tactile (-1 FB); (iii) simultaneous deprivation of two feedbacks, either visual and auditory, or visual and tactile, or auditory and tactile (-2 FB); (iv) simultaneous deprivation of three feedbacks, visual, auditory and tactile (-3 FB). Participants were deprived of visual and auditory feedbacks using a sleeping mask and ear defenders, respectively. The tactile feedback was prevented by the means of a removable striking surface at the place where the index finger tapped (“air tapping,” e.g., Aschersleben and Prinz, 1997). Each participant performed three tapping trials in the assigned conditions. As mentioned above, none of the visual, auditory or tactile deprivations should alter tapping performance (Aschersleben and Prinz, 1995, 1997; Repp and Su, 2013), and no study to our knowledge conveys strong assumptions about any differential effect of these conditions on the temporal structure of tapping. Nevertheless, rather than arbitrarily removing one of the three feedbacks for each participant or for a whole group, participants of the −1 FB group performed one trial in each of the visual, auditory and tactile feedback deprivation conditions in a random order. Likewise, participants of the −2 FB group performed one trial in each of the visual-auditory, visual-tactile, and auditory-tactile deprivation conditions in a random order. Participants of the Control and −3 FB groups performed three times the same.

### The tapping task

The experiment was conducted in a quiet room. Participants were sitting comfortably on an adjustable chair, with their dominant side forearm and palm of the hand resting on a customized plinth (570 × 160 × 50 mm) on a table in front of them. Subjects realized a tapping task according to a conventional synchronization-continuation paradigm (Wing and Kristofferson, 1973; Vergotte et al., 2017): during the initial synchronization phase, the tempo was prescribed by a PC-driven auditory metronome delivering 20 signals at a frequency of 1.5 Hz (0.666 s inter-tap intervals), known as a comfortable tapping frequency (Fraisse, 1966; Torre and Delignières, 2008). Once the metronome stopped, participants had to continue tapping by maintaining the prescribed tempo as accurately and regularly as possible for the whole trial duration. The duration of each trial was set to 6 min 40 s so as to ensure a sufficient number of inter-tap intervals to be submitted to subsequent fractal analysis (Delignieres et al., 2006; Eke et al., 2012). Between each of the three trials, participants had a 2-min rest.

### Data collection

#### Tapping performance

Movements of the index finger were captured using a single-axis accelerometer (15 × 15 mm) fixed on the nail so as to minimize possible device-induced sensorial feedbacks. Acceleration data were collected using a Labjack U12 device and stored via its software (LJStream v1.07). The sampling rate was 300 Hz.

#### Functional near-infrared spectroscopy measurements

**Figure 1:**
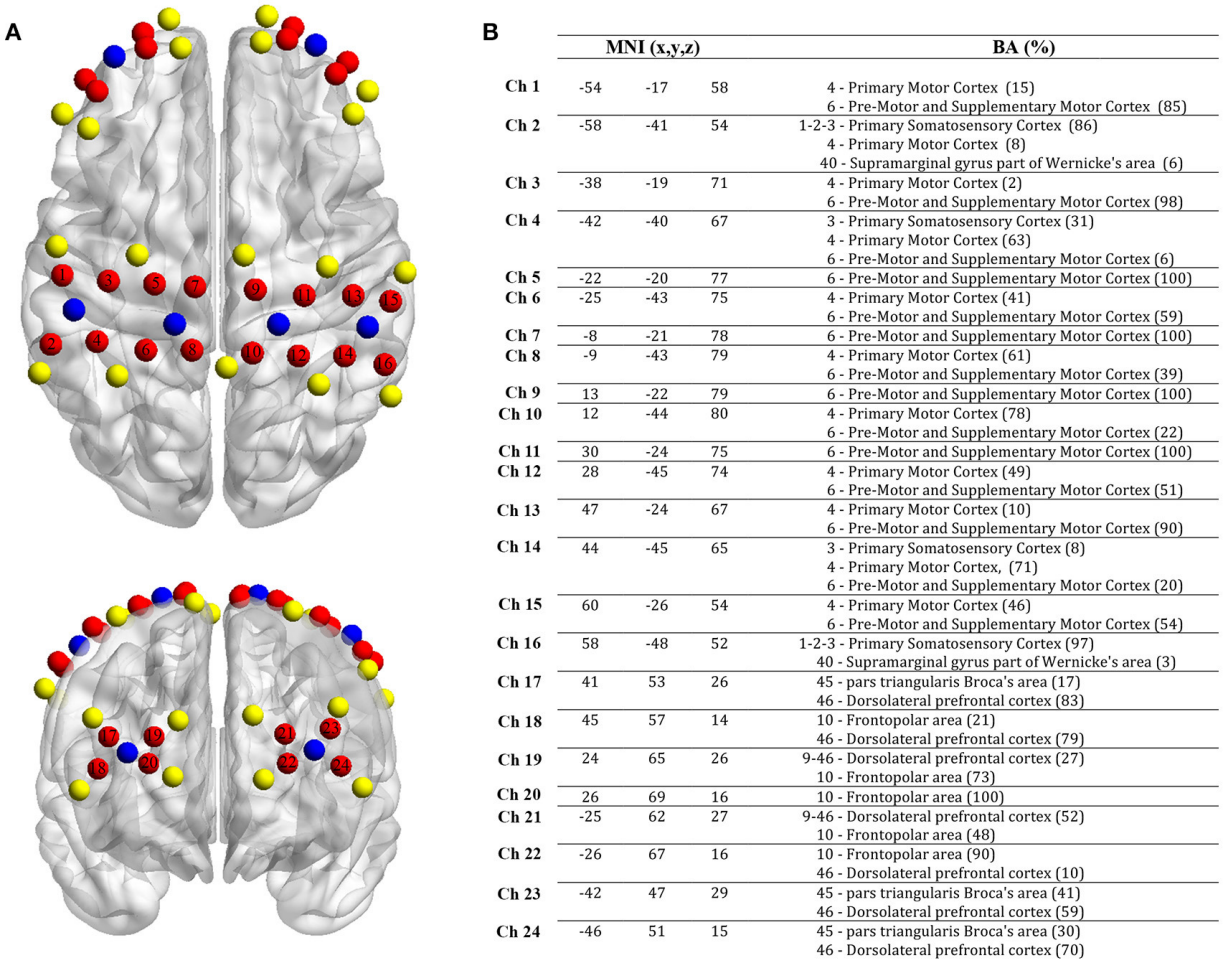
Localization of fNIRS probes, channels, MNI coordinates and Brodmann correspondences. **(A)** Yellow: transmitters, blue: detectors and red: channels. **(B)** MNI coordinates for each channel (*n* = 24) with x, y, and z coordinates. On the right, Brodmann area correspondence (number, name and %) extracted from the NIRS-SPM toolbox (NFRI function).

Hemodynamic changes in the cortex during the tapping tasks were measured by two synchronized continuous waves (CW) multi-channel functional near infrared spectroscopy (fNIRS) devices (Oxymon MkIII and Octamon, Artinis Medical Systems, The Netherlands) with a sampling rate of 10 Hz. fNIRS is an optical method using near-infrared light to measure relative changes of oxyhemoglobin (O_2_Hb) and deoxyhemoglobin (HHb) in the cortex (Scholkmann et al., 2014). In the present study, a customized cap was used to place beside the vertex (Cz) a 16-channels array on three regions of interest [premotor cortex (PMC), primary motor cortex (M1) and supplementary motor cortex (SMA)] on both hemispheres. Another 8-channel array was placed on the prefrontal cortex [PFC, Nazion (Nz) was the reference point]. Due to different sensibility of light penetration among brain regions using fNIRS (Brigadoi and Cooper, 2015), the inter-probe distance was fixed at 30 mm for M1, PMC and SMA, and 35 mm for PFC. After positioning all we used a 3D-digitizer (Fastrack, Polhemus, United States) to collect the location of each probe for each subject. NFRI function (Singh et al., 2005) included in the NIRS-SPM toolbox (Ye et al., 2009) was used to extract the Montreal Neurological Institute coordinates (MNI). The positioning of the 24 channels (MNI coordinates and Brodmann area correspondences) can be seen in Figure 1.

### Data analysis

#### Motor variability analysis

##### Preprocessing of tapping data

Raw acceleration data were first low-pass filtered using a Butterworth zero-phase digital filter (Frequency = 15 Hz). Then, a MATLAB in-house script (MATLAB 2014b, The MathWorks) for peak detection was used to extract the onsets of the subsequent finger taps. Series of inter-tap intervals (ITI) were then computed as the differences between subsequent tap times. For each trial, the first twenty ITI (corresponding to the synchronization phase) were discarded, and series of 512 ITI in the continuation phase were retained for further analyses. For each ITI series, we computed the typical performance variables used in tapping studies (Billon et al., 1996; Repp and Su, 2013), namely the mean, the coefficient of variation (CV) and the linear drift over the trial duration.

##### Characterizing fractal properties of inter-tap interval series

Fractal time series are basically characterized by fluctuations with scale invariant structure [i.e., obeying a power law distribution *X(ct)* = *c*^*H*^*X(t)*, where *X* is the signal, *c* is a constant, *H* is the fractal exponent] and temporal long-range correlations (meaning the autocorrelation function of the time series decays as a power-law without falling to zero). To analyze the fractal properties of ITI series, we used the Multifractal Detrended Fluctuation Analysis (Ivanov et al., 1999; Kantelhardt et al., 2002; MFDFA, Ihlen, 2012). MFDFA is derived from the original Detrended Fluctuation Analysis (DFA), which estimates the monofractal properties of a time series (Peng et al., 1995). In short, DFA exploits the diffusion properties of the time series, analyzing the relationship between the average amplitude of fluctuations and the size of the observation window within which these fluctuations are measured. For fractal series, a power-relationship characterized by the monofractal exponent α ε [0, 2] is expected: in particular, for α = 0.5 the series is white noise, for α = 1, the series is so-called *1/f noise*, and for 0.5 < α < 1 the series is considered stationary and containing persistent long-range correlations. By yielding a single fractal exponent (α) characterizing the average fractal properties of a time series, the DFA assumes that the fractal properties are homogeneous over all scales of the entire time series.

However, instead of being characterized by a single homogeneous fractal exponent, time series of bio-behavioral variables are often characterized by an inhomogeneous distribution of variability (intermittent fluctuations). The fractal exponent may vary over time scales: the series is actually characterized by multiple fractal exponents (Ihlen and Vereijken, 2010) and with this viewpoint the MFDFA was developed (Kantelhardt et al., 2002; Ihlen, 2012). Since we hypothesized that the system's adaptations to imposed task constraints would be expressed through the variety of fractal properties in ITI series, we opted for MFDFA analysis. MFDFA basically uses the same steps as DFA, but the average amplitude of the fluctuations is calculated using *q*^*th*^ order fluctuation function, with *q* varying from−10 to +10 in steps of 0.5, whereas DFA computes the amplitude of fluctuations only for *q* = 2. In brief, the time series *x(i)* is first integrated into *X(k)*, and divided into *N*_*n*_ adjacent segments of length *n*. Within each segment (*s* = 1, …, *N*_*n*_) the local trend is then subtracted from *X(k)*. So, the amplitude of fluctuations is computed for each detrended segment according to:

(1) $$\begin{array}{l} F^{2}\left(n,s\right)=\frac{1}{n}\overset{sn}{\underset{k\;=\;\left(s-1\right)n+1}{\sum }}{\left[X\left(k\right)-X_{n,s}\left(k\right)\right]}^{2} \end{array}$$

The variance is then averaged over all segments to obtain the *q*^*th*^ order fluctuation function:

(2) $$\begin{array}{l} F_{q}\left(n\right)={\left\{\frac{1}{N_{n}}\overset{N_{n}}{\underset{s\;=\;1}{\sum }}{\left[F^{2}\left(n,s\right)\right]}^{q/2}\right\}}^{1/q} \end{array}$$

If the series *x(i)* presents fractal properties, the generalized Hurst exponent *h(q)* is given by:

(3) $$\begin{array}{l} F_{q}\left(n\right)\propto n^{h\left(q\right)} \end{array}$$

**Figure 2:**
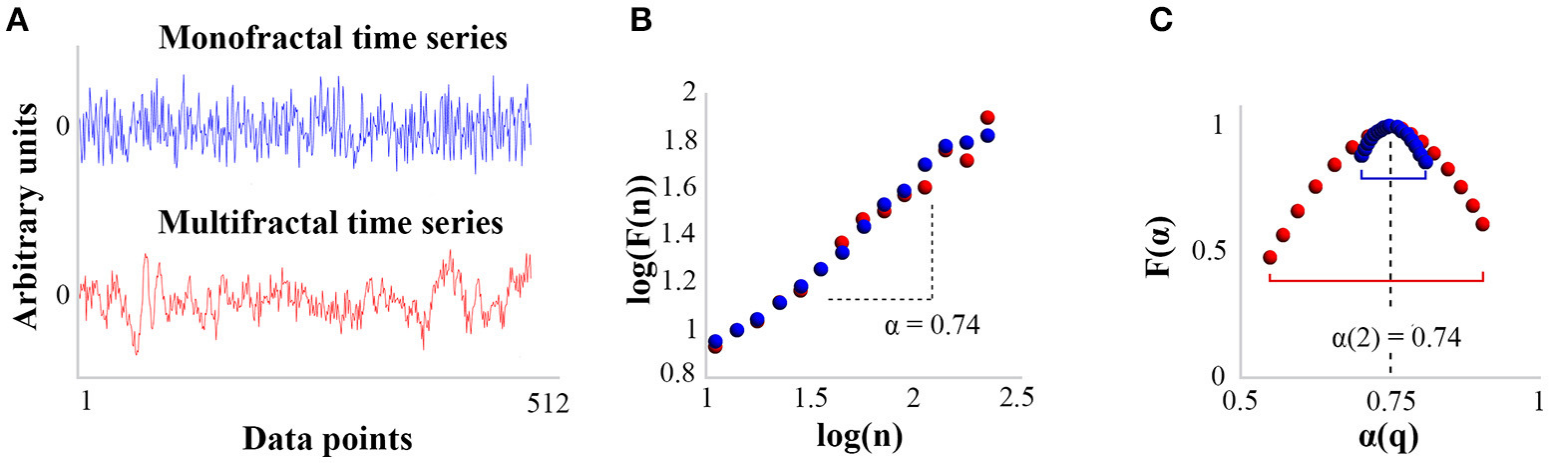
Distinction between DFA and MFDFA analysis for mono and multifractal time series. **(A)** Two experimental time series of 512 pts: in blue, the time series is closely mono-fractal; in red, the time series is multi-fractal. The Y-axis displays an arbitrary unit centered to zero. **(B)** Results yielded by DFA for the two time series. The plot shows the size of fluctuations *F(n)* as a function of the size *n* of observation windows in bi-logarithmic coordinates. The monofractal exponent α is given by the slope of log(*F(n)*) vs. log*(n)*. According to the plot, both time series present long-range correlations and are characterized by the same monofractal exponent (α = 0.74). **(C)** Multifractal spectra for the two time series. The right-hand side of the spectrum accounts for the influence of large-amplitude fluctuations (*q* positive), and the left-hand side accounts for the influence of low amplitude fluctuations (*q* negative). The width of the multifractal spectrum is then calculated by the difference α*(q)*_*max*_ – α*(q)*_*min*_. Comparison of plots **(B,C)** shows that while both time series present globally the same monofractal exponent, the blue series is close to monofractal whereas the red one is clearly multifractal.

According to Kantelhardt et al. (2002), the result of MFDFA can then be converted into the classical multifractal formulation using simple transformations, to be finally summarized by the multifractal spectrum representing *F(*α*)* as a function of α*(q)*, where *F(*α*)* is the fractal dimension (or dimension of the subset of the series that is characterized by α), and α is the Hölder (or singularity) exponent (see Figure 2). Our variable of interest is the width of the multifractal spectrum (MF-Width), meaning the range between the minimum and maximum exponents α*(q)* characterizing the time series, which represents the degree of multifractality. Figure 2 illustrates the distinction between mono and multifractal properties of two experimental time series as assessed by DFA and MFDFA.

#### Brain connectivity analysis

##### fNIRS preprocessing

A common approach as described in Huppert et al. (2009) was used to obtain O_2_Hb and HHb concentration changes. We extracted 6 min of raw (light intensity) data after the end of the metronome using the ARTINIS software (Oxysoft v3.0.95). Data were then uploaded in MATLAB. We first converted intensity data to optical density (OD). Then we applied the moving standard deviation and spline interpolation methods (SDThresh = 20, AMPThresh = 0.5, tMotion = 0.5 s, tMask = 2 s and p = 0.99; Scholkmann et al., 2010), combined with wavelet artifact correction (iqr = 0.1; Molavi and Dumont, 2012) as recommended in Cooper et al. (2012) to remove possible head motion artifacts. To retrieve the relative concentration changes (expressed in μM) of O_2_Hb and HHb, we applied the modified Beer-Lambert law (Kocsis et al., 2006) on OD data, by including an age-dependent constant differential path length factor (4.99 + 0.067 × Age^0.814^). The presence of a strong cardiac oscillation (frequency peak around 1 Hz) in the power spectrum of O_2_Hb signal indicates a good contact between the optical probe and the scalp (Themelis et al., 2007). 6.25% of all channels analyzed did not satisfy this condition and were discarded. For subsequent analysis, a band pass zero-phase digital filter (4th order Butterworth, cut-off frequency [0.009 0.08]) was used to remove physiological noise like cardiac, respiratory, Mayer waves and very low frequencies (Scholkmann et al., 2014). A linear detrending was then used to remove possible slow drifts.

##### Functional connectivity analysis

In the line of assessing functional network connectivity free from the constraint of neuroanatomical a-priori assumptions, the most commonly used method is based on the bivariate Pearson's correlation analysis (Biswal et al., 1995): it consists in computing the statistical dependency between two or more time series to explore the influence that one region of interest exerts on others (seed based correlation analysis), or in computing all possible connections at the level of the entire brain (whole brain correlation analysis), at rest or during a task (Medvedev, 2014).

**Figure 3:**
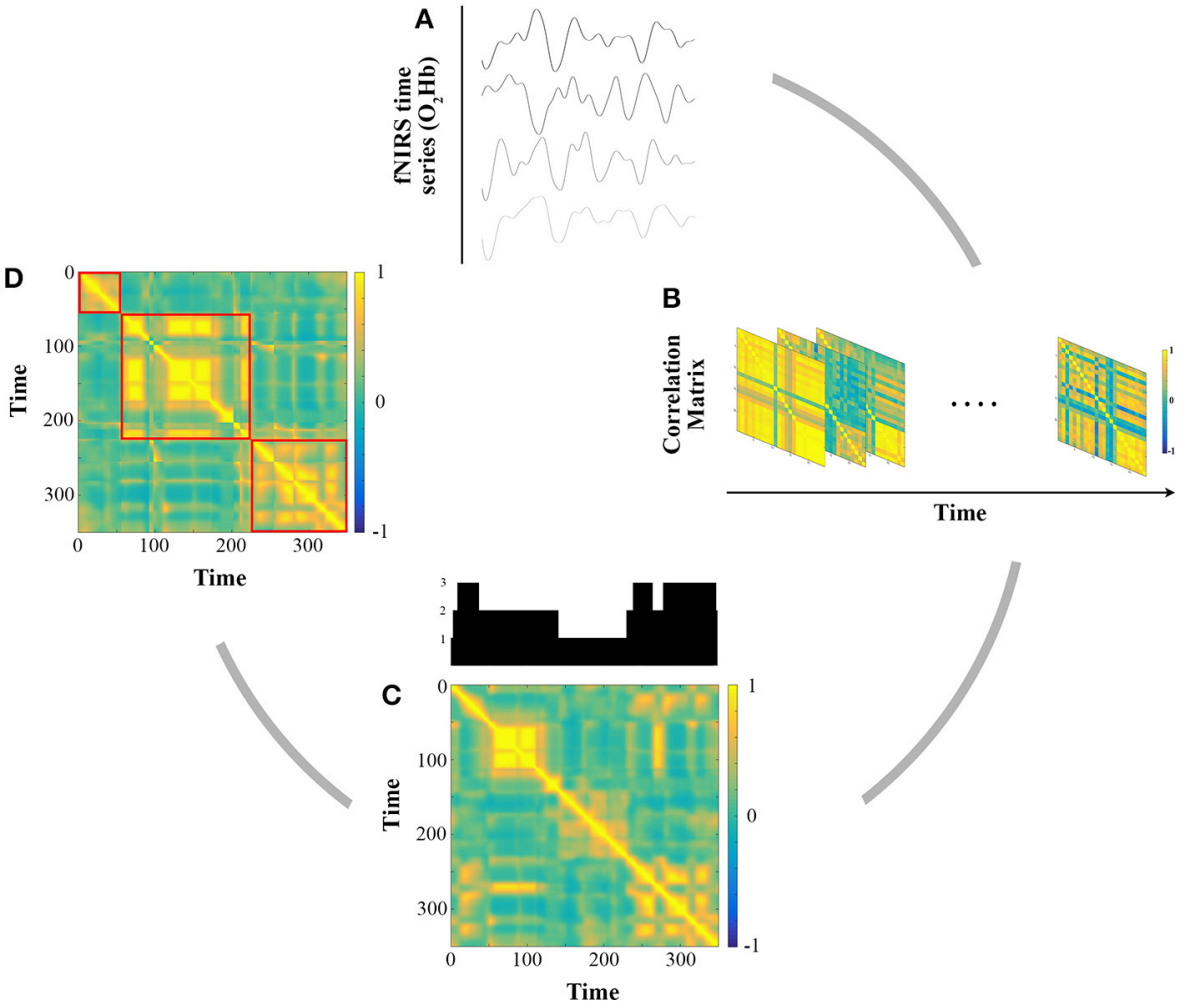
Illustration of the functional connectivity analysis for one representative subject. **(A)** Extraction of O_2_Hb fNIRS time series for all channels after preprocessing and band pass filtering (cut off frequency [0.08 0.009]). **(B)** Sliding window Pearson's correlation analysis for window sizes of 30, 75, and 120 s. **(C)** Grand average correlation analysis between each matrix. The upper plot shows communities detected for 360 s (3 communities in this example). **(D)** Grand average matrix after putting in the order of community. Red squares delimit each community.

Then an *N* × *N* adjacency matrix was constructed, reflecting the strength of the correlation between each time series. However, different studies applying such analyses have implicitly considered that patterns of connectivity were stationary and computed an average matrix over the whole scanning period. Instead, to assess the dynamic functional connectivity (dFC) between the present 24 fNIRS channels, we used a sliding window correlation analysis as proposed in the literature (Hutchison et al., 2013). For each subject, this method yielded a number *n* of matrices depending on the window size and a shift (in samples), summarizing the evolution of all connections between channels over time. As there is no consensus in the literature we used three widespread window sizes (30, 75, and 120 s; Hutchison et al., 2013) and a shift of 1 sample (100 ms). Figure 3 illustrates the pipeline for these analyses.

##### Modularity analysis

Once obtained the time evolution for all connections, one of the main challenges in dFC analysis is to classify the multiple networks obtained with reliable metrics (Fallani et al., 2014). A network is a collection of nodes (vertices) and links (edges). All networks are represented mathematically through their connectivity (adjacency) matrices. Rows and columns correspond to nodes and entries denote links that are weighted. Based on the graph theory analysis, one of the relevant methods to extract the number of different communities involved during the task is the modularity analysis (Newman, 2006; Rubinov and Sporns, 2010). Modularity quantifies the degree to which the network may be subdivided into delineated and no overlapping groups. In other words, modularity reflects strong links within each community and weaker links between communities. The Modularity (*Q*) for a partition in communities *m* = *[m1, …, mn]* of a weighted undirected graph is defined (Watts, 2004; Newman, 2006; Rubinov and Sporns, 2010) as:

(4) $$\begin{array}{l} Q_{\left(m1,\;\ldots ,mn\right)}^{w}=\frac{1}{l^{w}}\sum_{i,j\;\in \;N}\left[w_{ij}-\frac{k_{i}^{w}k_{j}^{w}}{l^{w}}\right]\delta_{mi,mj} \end{array}$$

where *w*_*ij*_ is the weight of the edge between node *i* and node *j*. The set of weights fits into a matrix *w* that represents the graph *G*. Here *w*_*ij*_ is the correlation between the row *i* and row *j* of functional O_2_Hb matrix. Rows and columns of the square matrix *G* are indexed by the nodes of *G* (that is the time index of O_2_Hb matrix). When connections are non-oriented (as in the present study) this matrix *G* is symmetric: weight *w*_*ij*_ = *w*_*ji*_ and *k*_*i*_ is the weight of vertex *i* that is the sum of *w*_*ij*_ for all vertices *j*. The number *l*^*w*^ is the total sum of weights. Modularity optimization was done based on the assumption that a graph partitioning is the difference between the number of edges within the partitions found and the number of expected edges at random between vertices of an equivalent degree distribution (Newman, 2006). In this formalism, the ratio $k_{i}^{w}k_{j}^{w}$/*l*^*w*^ gives the null model, that is the probability that a random edge with a random weight *w*_*ij*_ joins the nodes *i* and *j* (Newman, 2006). Nodes of *G* are partitioned between the sets *m1,…,mn*. So, *m*_*i*_ is the set of the actual partition that contains vertex *i*. The δ _(*mi, mj*)_ (delta of Dirac) function for given vertices *i* and *j* takes the value 1 if *i* and *j* are in the same subset of the partition (that is *m*_*i*_ = *m*_*j*_), and 0 otherwise. Importantly, in our study we used the modularity analysis across all time steps and not for each graph. We then considered that distinct community detected should reflect different network organization without extracting the exact topological organization. We determined the communities in each of these graphs by the algorithm that maximizes the modularity (see Equation 4) from the Brain Connectivity Toolbox (Rubinov and Sporns, 2010).

#### Statistical analysis

After normality testing (Lilliefors test), between-group differences were tested using one-way ANOVA on the three tapping performance variables (mean, CV and drift of ITI series), and on MF-Width with respect to our main hypothesis. Secondarily we also checked for any between-group difference in the monofractal exponent (α). We used Kruskal Wallis analysis, as the data were not normally distributed for the three sliding window sizes of community detection analysis. We used Spearman's correlation between the number of networks detected for each sliding window size (30, 75, and 120 s) and MF-Width of the tapping series.

## Results

### Tapping performance

**Figure 4:**
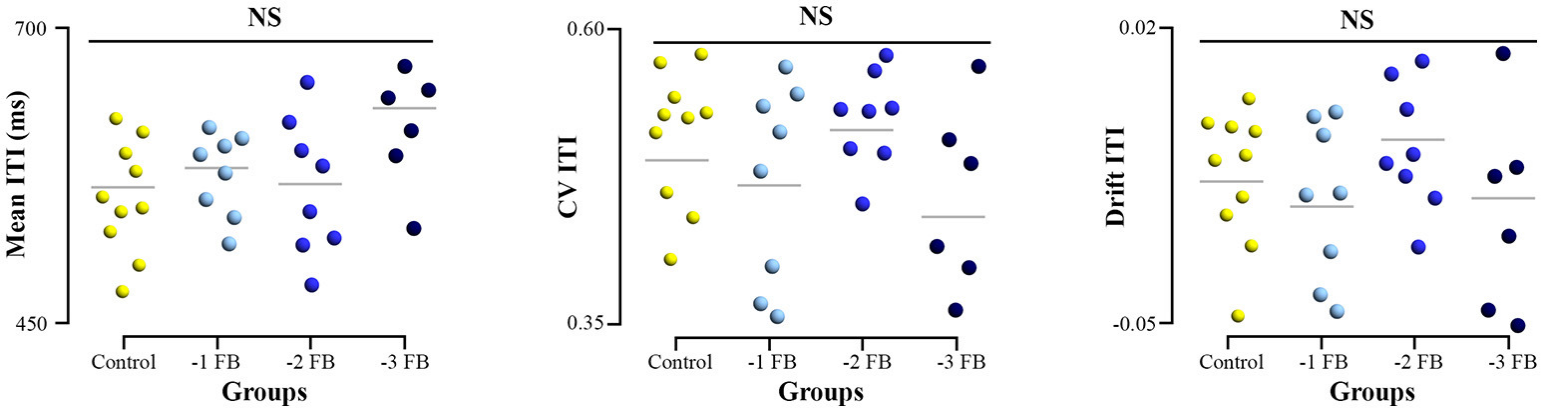
Dotplot of tapping performance for the four groups. Left, mean inter-tap intervals (ITI) produced. Middle, Coefficient of variation (CV) of ITI series. Right, drift of ITI series during the task. Error bars represent standard error.

All samples of the tapping performance variables (mean, CV, and drift) were normally distributed. Our experimental design was thought to impose different levels of constraints to the subjects without inducing differences in tapping performance. The ANOVA applied to the performance showed no significant difference between groups for all variables [mean: *F*_(3, 28)_ = 1.519; *p* = 0.230; η^2^ = 0.136, CV: *F*_(3, 28)_ = 2.316; *p* = 0.523; η^2^ = 0.045, drift: *F*_(3, 28)_ = 0.634; *p* = 0.594; η^2^ = 0.022, Figure 4].

### Multi-fractal properties of tapping series

**Figure 5:**
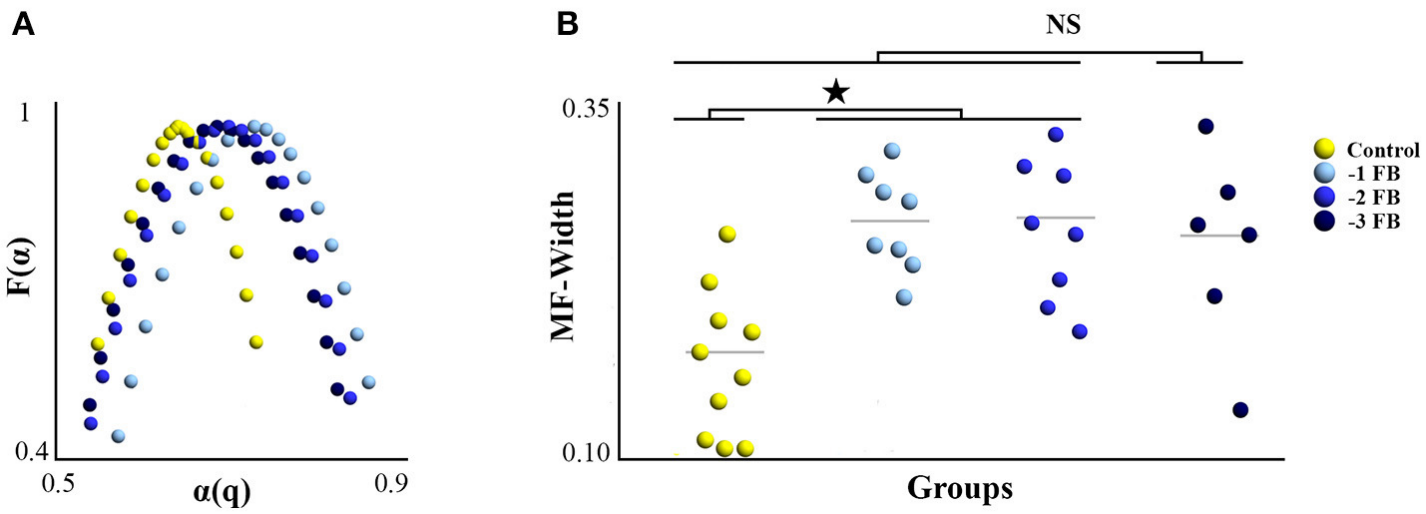
Degree of multifractality of ITI series (MF-Width) for the four experimental groups. **(A)** Average multifractal spectrum for each group. **(B)** Dotplot MF-Width for the four experimental groups; gray horizontal line represent the mean. Star reflects the significant difference at *p* < 0.05.

MF-Width samples were normally distributed after log-normal correction. The one-way ANOVA revealed a significant group effect [*F*_(3, 28)_ = 2.822; *p* = 0.044; η^2^ = 0.253]. LSD Fisher *post-hoc* showed differences between the control group and the −1 FB and −2 FB groups (*p* = 0.012 and *p* = 0.021, respectively). Figure 5 summarizes the results obtained for the multifractal properties of tapping time series. Monofractal exponents (α) were normally distributed, and the one-way ANOVA did not show any significant difference between groups [*F*_(3, 28)_ = 0.845; *p* = 0.473; η^2^ = 0.071].

### Modularity analysis

**Figure 6:**
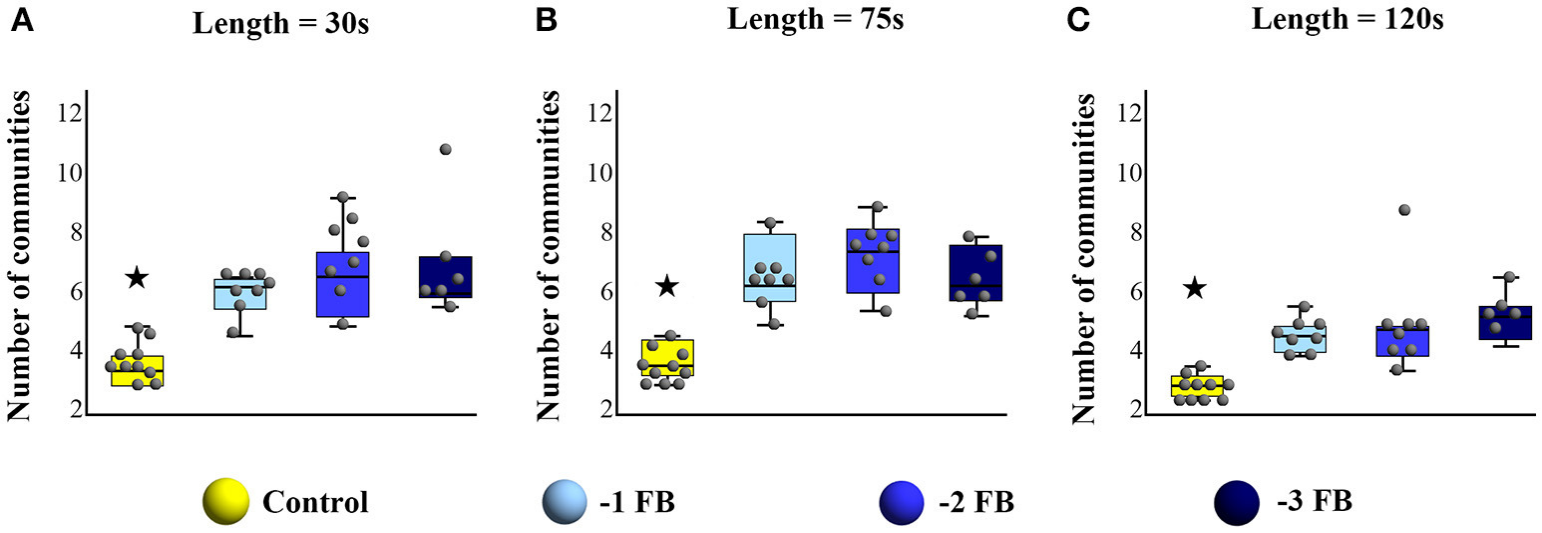
Box plots with median, quartiles, and individual dots for the number of communities detected during the task for the four groups (Control,−1FB,−2 FB, and−3 FB), for **(A)** sliding window of 30 s, **(B)** sliding window of 75 s, and **(C)** sliding window of 120 s. Stars highlight significant difference at *p* < 0.05.

For all considered sliding window sizes, Kruskal Wallis test showed significant differences between Control and −1 FB, − 2 FB and − 3 FB groups [for 30 s: *H*_(3)_ = 18.7, *p* = 0.003; η^2^ = 0.561; for 75 s: *H*_(3)_ = 18.5, *p* = 0.001; η^2^ = 0.554; for 120 s: *H*_(3)_ = 18.9; *p* = 0.003; η^2^ = 0.568]. All corrected *p*-values for multiple comparisons are reported in Table 1. Results for each window size are shown in Figure 6.

**Table 1:** Corrected *p*-values of Kruskal Wallis analysis for each sliding window size.

	**Window size**		
	**30 s**	**75 s**	**120 s**
Control/−1 FB	<**0.009**	<**0.04**	<**0.03**
Control/−2 FB	<**0.003**	<**0.0002**	<**0.02**
Control/−3 FB	<**0.006**	<**0.02**	<**0.01**
−1 FB/−2 FB	1	1	1
−1 FB/−3 FB	1	1	1
−2 FB/−3 FB	1	1	1

*Significant differences are in bold*.

### Relationship between modularity in the brain and fractal properties in behavior

**Figure 7:**
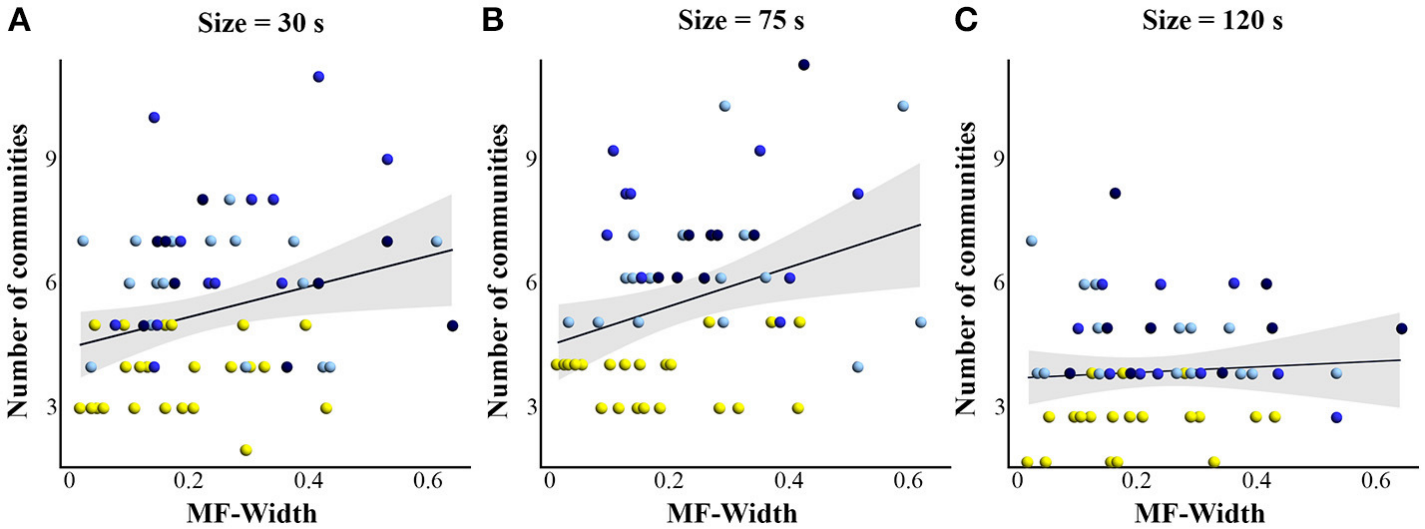
Scatterplots showing the correlation (Spearman's correlation) between the multifractal properties (MF-Width) and the number of communities detected with modularity analysis, for **(A)** window size = 30 s, **(B)** window size = 75 s, and **(C)** window size = 120 s. The correlation is significant for 30 and 75 s windows. Yellow = control group, light blue = −1 FB, blue = −2 FB, and dark blue = −3 FB group.

With regard to our main hypothesis, results showed a significant correlation (Figure 7) between MF-Width in tapping series and the number of brain networks detected for window sizes 30 s (rho = 0.277; *p* = 0.028) and 75 s (rho = 0.275; *p* = 0.038). However no significant correlation was found for window size 120 s (rho = 0.086; *p* = 0.526).

## Discussion

The present study aimed to establish a relationship between connectivity patterns underlying adaptation at the brain level and fractal properties as their dynamic signature in the behavior. We hypothesized that the number of brain networks involved in the task and the multifractal properties of the tapping series would evolve jointly, as a function of different conditions of feedback deprivation. We found that (i) the degree of multifractality (MF-Width) increased significantly in groups where feedbacks were suppressed as compared to the control group; (ii) the number of networks involved during the task was higher for groups with feedback deprivation than for the control group; and (iii) MF-Width and the number of networks involved in the task were correlated for sliding windows of 30 and 75 s. After discussing the suitability of the experimental design, we consider some notable implications of our results at the behavioral and brain levels, respectively, before focusing more specifically on the brain-behavior relationship.

### Suitability of the experimental design

We considered the general definition of adaptability as the capacity to maintain a given function or performance despite changing constraints (De Wolf and Holvoet, 2005), also referred to as robustness (Whitacre, 2010). In this line, the experimental paradigm was required in order to impose different experimental constraints while leaving the global level of task performance substantially close: the system was thus deemed to handle adaptations—notably reorganization in brain—allowing for sustained performance. To meet these requirements, we used a finger-tapping task. A major advantage of such task was to allow for a simple manipulation of the amount of sensory feedbacks available (–1 FB,−2 FB,−3 FB), while overall tapping performance has previously been shown insensitive to feedback manipulations (Aschersleben and Prinz, 1995, 1997; Repp and Su, 2013). That is, in the present study feedback manipulation has merely constituted a means to constrain the system and induce putative internal adaptation/reorganization, without any specific hypothesis as regards the sensory modalities. In this respect, our results are congruent with the literature (Aschersleben and Prinz, 1997), as we observed no significant differences between conditions of feedback deprivation in any of the three variables commonly characterizing tapping performance (mean ITI, CV and drift; Figure 4). Moreover, such tapping task has previously been shown to entail fractal properties in the ITI series produced (Lemoine et al., 2006; Torre and Delignières, 2008). Our present results on the monofractal exponent α are also in agreement with the literature in this respect (α = 0.75 ± 0.13 all groups taken together, without significant differences between groups).

As regards the experimental design, we opted for an independent group design rather than repeated measures. Although this methodological choice obviously entailed limitations of sample sizes for each group, we deemed it preferable given the lengthy duration of tapping trials required for reliable fractal analysis (Delignieres et al., 2006; Vaz et al., 2017). Indeed, we aimed to observe the effect of adaptations due to feedback deprivation, which implied avoiding as much as possible any putative effects of weariness and attentional fluctuations that may also alter the fractal properties of tapping series (Damouras et al., 2010). Finally, in contrast to previous studies we here investigated a motor task with adaptations being experimentally induced by different levels of task constraints. In return, this approach implied some *a priori* uncertainty as regards the precise effect of experimental constraints especially on brain connectivity, rather than *a priori* controlled variations as possible in simulation studies for example. All in all, the consistency of our results with previous literature leads us to consider the experimental design suitable, and following results with reasonable confidence.

### Multifractal properties reflect adaptations underlying unchanged performance

Further gain of precision in appraising the functional significance of fractal properties in behavioral variables is a still-open challenge. A significant body of literature has converged to the general idea that mono and multifractal properties are a hallmark of the adaptability of biological systems (Goldberger et al., 2002; Lipsitz, 2002). However, such conclusions mostly originate from indirect cross-sectional observations revealing loss of fractal properties with aging, pathology, or different conditions of functional impairment that are generally associated with loss of adaptability (Manor et al., 2010; Manor and Lipsitz, 2013). Though adaptability (or loss of adaptability) may indeed constitute a common denominator, several potentially confounding effects, including effective adaptations to achieve task performance despite functional impairment, might actually be the cause of altered fractal properties (Dingwell and Cusumano, 2010).

Our present results show significant variations of multifractal properties as a function of feedback deprivation imposed to the system (Figure 5) without significant functional decrement (Figure 4), which does not appear directly relevant to the issue of adaptability. At a first glance, this result may appear congruent with previous studies showing an alteration of monofractal properties as a function of the involvement of sensorial feedbacks in task performance (Slifkin and Eder, 2012, 2014): it has indeed been proposed that weaker monofractal properties may be due to tighter sensorimotor control mechanism exerted on task-relevant variables (Dingwell and Cusumano, 2010; Warlop et al., 2013). However, we observed that the degree of multifractality in tapping series increased in feedback deprivation conditions compared to the control group. Mono and multifractal properties do not capture the same features of time series: whereas monofractal properties summarize a homogeneous scaling behavior over the whole time series, multifractal analyses assess the possibly inhomogeneous scaling regimes present in the series, and capture the amount of intermittent changes in the systems/subjects functioning modes (Ihlen and Vereijken, 2010). Thus, this result suggests an increasing involvement of different modes of regulation to achieve unchanged performance despite the imposed experimental constraints. Accordingly, we support the idea that rather than globally reflecting the adaptability of complex biological systems, changes in multifractal properties reflect effective adaptations underlying invariance of functional outcome.

In this line, characterizing multifractal properties in macroscale variables may constitute a fine-grained analysis to uncover masked adaptations underlying goal achievement. From a broader perspective, disentangling adaptability and effective adaptation actually constitutes a major challenge, as both come necessarily together to a certain extent (Ulanowicz, 2002). Combining analysis at the task-relevant observation level (e.g., the level of motor performance) and an assessment of the correlates occurring at underlying observation levels (e.g., the level of brain dynamics) may contribute to this end.

### Changes in brain modularity reflect functional adaptation to constraints

In this study, we hypothesized that the variety and intermittency of functional connectivity patterns would be influenced by different conditions of privation of sensorial feedbacks. Our results show that dynamical reorganizations of the brain network yielded multiple networks that were intermittently involved during task performance (Figures 3, 6), and that the number of different networks involved depends on the experimental group, i.e., on the feedbacks subjects were deprived or not. The literature studying brain networks involved in a task has generally considered that for a given function or performance the functional organization of the brain is stable over time. Accordingly, the purpose of investigations has often been to extract the typical network engaged in a given task, using a number of computational methods (Biswal et al., 1995; Witt et al., 2008). Nevertheless, another part of the literature studying the dynamic properties of functional networks in resting state has showed that the modular organization of the brain evolves within the scanning period (Chang and Glover, 2010; Hutchison et al., 2013; Fallani et al., 2014) and that such natural fluctuations are likely to support the ability of quick adaptive responses (Deco et al., 2011). Previous studies using resting paradigm do not enable to reveal the actual implementation of such adaptability during a sensorimotor task. These results complement previous literature insofar as they show that the variety of brain networks that are involved in a single task depends on the experimental constraints imposed to the subjects. More precisely, the counter-intuitive character of these results (i.e., increased number of networks with decreasing feedbacks, Figure 6) could be explained by the fact that, under constraints, the brain navigates between numerous networks to find out the solution enabling achievement of its level of performance (Kelso et al., 2013; Tognoli and Kelso, 2014). Such explanation seems consistent with previous studies showing novel recruitment of cortical areas under conditions of chronic sensorial deprivation (blindness, deafness, Merabet and Pascual-Leone, 2010), although adaptation to transient experimental manipulations is not directly comparable with lifelong alterations.

From a broader perspective, considering that the brain possesses degenerate properties (i.e., multiple networks could perform the same function with some of them being possibly latent, Edelman and Gally, 2001), the networks involved in a given function or performance can hardly be grasped in a comprehensive way without imposing internal and/or external constraints so as to induce variation in connectivity patterns (Price and Friston, 2002). This idea was initially developed in theoretical papers, and few experimental tasks actually allow imposing constraints without changing the motor performance. Electroencephalography, functional magnetic resonance imagery and fNIRS studies (Nedelko et al., 2010; Leff et al., 2011; Muthuraman et al., 2012) showed that the sensorimotor network (e.g., M1, PMC, and PFC) is engaged in a simple short finger-tapping task and is supposed to reflect sensory integration, motor initiation and production. Conversely, our results suggest the existence of multiple networks that allow for the carrying out of a tapping task over time. Moreover, there is no single network dedicated specifically to tapping independently of the different conditions under which tapping is to be performed. However, these findings need to be examined with caution due to some methodological consideration. In this study, we used modularity analysis (Newman, 2006; Sporns, 2012; or community detection, Sporns and Betzel, 2016) at the macro scale level (between networks) and not on each network. One can hypothesize that the latter analysis would make it possible to highlight similar clusters of sub-networks linked in different ways. In particular, it has recently been shown that dynamic connectivity between different brain regions is not only dependent on the regions involved, but also on the interconnections between multiple EEG frequency bands (Liu et al., 2015). Future investigations using EEG combined with fNIRS would allow to better understand the dynamic functional organization of the brain, and the role of multifrequency connections in network coupling. It has been proposed that the modular organization of the brain is subtended by a relatively rigid network composed of nodes distributed in each sub-module (Sporns, 2013). Nevertheless, although the origin of temporal fluctuations in dFC estimates remains largely unknown, sliding window analysis was shown as a promising method to highlight dynamic connectivity in multiple neuroimaging methods. As the optimal window size to compute correlation coefficient is still under debate (Hutchison et al., 2013), we used three-window sizes (30, 75, and 120 s, see Figure 7) to be confident in the results obtained. We found a strong statistical difference between the control group and other groups independently of the window size. This confirms our hypothesis and this allows us to confirm that our results are not dependent on the window size chosen (e.g., Hutchison et al., 2013; Hindriks et al., 2016). An additional step of our promising results would be to extract the characteristics of the different networks implemented with more fine-grained tools like those proposed in fMRI (Bassett and Bullmore, 2006; Bassett and Gazzaniga, 2011; Papo et al., 2014).

### Bridging the gap between brain and behavior

The literature has mostly been studying the dynamics of cerebral networks on one hand, and the temporal structure of behavioral variability on the other hand, though both communities share key concepts coming with the complex system approach (Bullmore et al., 2009; Werner, 2010; Whitacre and Bender, 2010; Sleimen-Malkoun et al., 2014). Thus, attempts to link these two approaches seem valuable (Price and Friston, 2002; Friston and Price, 2011). In the present study, we provide novel evidence that the number of networks involved during a motor task in four experimental conditions significantly correlates with the degree of multifractality found in the sensorimotor outcome. This correlation was obtained for two of the three window sizes used (30 and 75 s). Previous literature has highlighted that the dynamics of functional connectivity increase with diminution of the window size, due to the non stationarity of Blood Oxygenation Level Dependent or fNIRS signals for short windows with an increase of transient nodes that were unobserved for large window size (Hutchison et al., 2013). Therefore it is not surprising that fewer networks were detected for our largest window (120 s). As a consequence the correlation between the number of networks and the multifractal properties of tapping series was low and not significant for the 120 s window as compared to the smaller windows.

Previous theoretical and simulation approaches had shown that degeneracy plays a central role in the link between complexity, adaptability, and robustness (Whitacre, 2010) and that degeneracy may underlie fractal properties in the outcome variables (Delignières and Marmelat, 2013). Our result provides experimental support highlighting the link between theoretically related properties across two different scales of observation, namely between degeneracy at the level of brain connectivity and measures of complexity at the level of behavior, both being considered tightly related with systems adaptability. As such this result may be of particular relevance for translational research, since a significant part of literature has proposed to assess the diagnostic and/or prognostic power of fractal properties in sensorimotor variables in neurodegenerative pathologies (e.g., Parkinson or Alzheimer diseases) conveying the strong but so far experimentally unproven assumption that alterations of the brain network would come out in the fractal properties of behavior. Consequently, we consider that (i) fractal properties in macroscale variables are (at least partly) dependent on the degenerate organization properties of the brain, and (ii) concomitant changes in network connectivity and multifractal properties in behavioral variability reflect (at least partly) effective adaptations underlying invariance of functional outcome.

Finally, the system's ability to adapt and effective adaptation go hand in hand (Ulanowicz, 2002), the first being a necessary condition for the latter, the latter in turn affecting the first. To be able to disentangle the brain and behavioral correlates of adaptability and adaptation is of importance seeing that evolution toward pathological states or advancing age often come along with a decreased ability to adapt, up to functional loss (Lipsitz, 2002; Manor et al., 2010; Stergiou et al., 2016). The joint analysis of motor variability and brain dynamics, as well as the use of an experimental paradigm that allows to gradually constraining the system so as to induce adaptations (maintenance of performance) up to the loss of further capacity to adapt (decrement of performance), may contribute to this end. Extending the present tapping paradigm may be appropriate in this view since, in contrast to visual, auditory or tactile feedbacks, further deprivation of proprioception has been shown to decrease tapping performance (Stenneken et al., 2006). Our present experimental design was not conceived such as to allow for investigation of putative differential effects among sensory modalities (e.g., auditory and visual cortex), and we limited ourselves to the assessment of sensorimotor and prefrontal regions. Future studies using a larger number of channels (whole brain) may examine in how far the networks dynamics underlying finger tapping are affected depending on the sensory modality suppressed.

## Conclusion

To what extent the multiple networks in the brain restructure with some distinctive properties of motor variability has remained unanswered so far. Both conceptual considerations and simulation approaches have provided strong indications for such relationship but experimental evidence has been lacking. Our present work evidences a significant correlation between the number of brain networks and the degree of multifractality in tapping. We believe that this finding constitutes a step further toward bridging the gap between the degenerate connectivity patterns at the brain level and the properties of variability at the behavioral level. We anticipate that future work, possibly combining simulation and experimental methods like multimodal neuroimaging, will provide means for larger and/or more fine-grained ranges of variation in the number of brain networks involved and the fractal properties of motor performance, so as to further consolidate our present findings.

## Author contributions

All authors contributed to this study. GV, KT, and SP conceived and designed the study. KT, MM, and SP provided funding. GV performed the experiments. GV, MM, and SJ analyzed the data. GV, KT, and SP wrote the paper and SJ and MM edited the manuscript.

### Conflict of interest statement

The authors declare that the research was conducted in the absence of any commercial or financial relationships that could be construed as a potential conflict of interest.
